# Exploring Applications of AI in the Crisis Line Sector: Protocol for a Scoping Review

**DOI:** 10.2196/95087

**Published:** 2026-08-13

**Authors:** Allison Crawford, Mackenzie Earle, Gisell Castillo, Nadia Nandlall, Mackenzie Hilton

**Affiliations:** 1 Centre for Addiction and Mental Health Toronto, ON Canada; 2 Department of Psychiatry University of Toronto Toronto, ON Canada

**Keywords:** AI, artificial intelligence, LLM, large language model, NLP, natural language processing, crisis lines, crisis helplines, suicide prevention

## Abstract

**Background:**

Crisis helplines are a vital component of a robust public health approach to suicide prevention as they are often free, accessible, and provide immediate support to individuals in distress. AI presents an opportunity for novel applications to support and improve crisis line services across a variety of functions, including assessing suicide risk, identifying issues, tracking responder behaviors, and providing prompts and reminders. However, the use of AI in the crisis sector also raises critical questions regarding safety, ethics, privacy, efficacy, feasibility, and acceptability among interest holders. The extent to which AI is currently being explored and implemented in crisis line contexts is unknown.

**Objective:**

The objective of this scoping review is to examine how AI is being used in crisis line services and to explore the perspectives of different interest holders regarding its application.

**Methods:**

This scoping review will be conducted in accordance with a validated methodological framework and reported using the PRISMA-ScR (Preferred Reporting Items for Systematic Reviews and Meta-Analyses extension for Scoping Reviews). A comprehensive search strategy will be developed with an experienced health sciences librarian and implemented in multiple databases, including Ovid MEDLINE, Embase, APA PsycInfo, CINAHL, IEEE Xplore, ACM Digital Library, and Web of Science. We will include primary research describing AI applications and implementation in the crisis line sector and studies exploring interest holder perspectives. Screening of titles and abstracts and of full texts will be performed independently and in duplicate following a screening calibration process. Disagreements will be resolved through discussion. Expert opinions will be sought as needed to make final determinations regarding included and excluded sources. A data extraction form will be created and piloted to capture information on study characteristics, study findings and outcomes, AI use cases, and considerations for various concepts relevant to crisis line services (eg, safety, trust, equity, ethics, and privacy). Extracted data will be synthesized using descriptive and narrative approaches.

**Results:**

As of June 2026, the search strategy has been finalized and translated across databases. Screening of 3380 unique records is ongoing and will conclude by September 2026. Results will be available by winter 2027.

**Conclusions:**

AI is changing public mental health by creating new opportunities to optimize interventions and enhance care. However, several implementation challenges remain regarding the use of AI applications in the crisis sector. This review is a timely and comprehensive exploration into the state of AI in the crisis sector and will identify existing knowledge and evidence gaps to help inform practice, policy, and future research. By systematically mapping current AI applications; interest holder perspectives; and how core domains such as safety, privacy, and equity are addressed, this review will provide a foundational evidence base to guide responsible, person-centered AI integration and a robust research agenda tailored to crisis line contexts.

**International Registered Report Identifier (IRRID):**

PRR1-10.2196/95087

## Introduction

### Background

Suicide is a major public health concern, with nearly 800,000 lives lost globally each year [1]. Crisis helplines are an evidence-based approach to suicide prevention that allow individuals in distress to call, text, or chat with a trained responder who provides immediate crisis support through empathy, active listening, collaborative problem-solving, and safety planning [2-4]. AI refers to computer systems capable of performing tasks that typically require human intelligence, such as reasoning, learning, problem-solving, and language processing [5]. The advent of AI applications and uses in mental health care—including machine learning (ML), natural language processing (NLP), and large language models (LLMs), and retrieval-augmented generation (RAG)—presents innovative opportunities to bolster and improve crisis line services. For example, systematic reviews on the use of AI to improve suicide risk assessments have found that AI performance is acceptable and holds high potential for supporting clinicians in accurately identifying people at risk of suicide [6,7]. Generative AI has been tested for its effectiveness in supporting clients undergoing cognitive behavioral therapy, with promising results [8]. Some research suggests that generative AI chatbots may help reduce mental health symptoms, though results have been inconsistent [9]. Others have begun exploring the use of AI to support therapist workflows [10]. A recent review of novel crisis line interventions also found that some crisis line services are exploring the use of AI to support various crisis line functions, including suicide risk assessment, issue identification, tracking responder behaviors, process reminders, and other applications [11].

At the same time, the introduction of AI into the crisis sector introduces important questions and considerations related to user and responder experience. One study on crisis chat user experiences found that chatters often questioned the authenticity of the responder by asking whether they were a human or a robot [12]. Another study found that crisis line service users and potential help seekers were fearful of technological advances leading to AI replacing human interactions during crisis line interactions [13]. Crisis line responders, while holding favorable views of AI’s potential in the sector also voiced concerns that AI-supported crisis line interactions may be experienced as dehumanizing [14]. Regardless of the AI use case, trust in AI-supported systems is paramount to any AI application.

Other critical issues that have been widely discussed include safety, privacy, legal, and the ethical implications of using AI in a highly sensitive environment that values the relational aspects of providing support [15,16]. The application of AI in health and mental health has raised concerns about informed consent, individual autonomy over data use, the potential risk and harms of generative AI “hallucinations,” errors and biases in predictions and clinical assessments (particularly for equity deserving groups), and the lack of transparency in explaining the processes that produce outputs [15,17-20]. The promise of AI in mental health and suicide prevention is, thus, tempered by these ethical and privacy challenges, with some researchers emphasizing the need to assess alignment with clinical goals and the readiness of mental health contexts to implement AI applications [21-24]. Concerns regarding the safety and well-being of those who engage with AI is paramount and has fortunately led to the development of guidance documents to aid in reducing potential harms, evaluative frameworks, predictive models that aim to account for social determinants of health, and recommendations for further research and AI model advancements [16-18,22,25].

However, questions remain regarding how effective AI-driven approaches are in enhancing crisis support; how they may influence the rapport between responders and service users; and what safeguards are required to ensure privacy, transparency, and fairness [16]. These questions also need to be addressed with full consideration of potential impacts on equity and whether AI use by crisis lines risks amplifying biases and prejudices or widening equity gaps [15].

There are also implementation questions regarding the feasibility, scalability, and sustainability of AI applications as successful integration of AI technologies depends on adequate technological infrastructure, education and training for crisis line staff, and clear guidance on appropriate use. To begin mapping out the state of AI use in the crisis line sector, we aim to use scoping review methodology to identify and describe relevant literature and identify knowledge gaps [26].

### Objective

The objective of this scoping review is to understand how AI is being used, implemented, and evaluated by crisis lines; the perspectives of different interest holders in the crisis sector on its application; and the associated challenges of using AI in the crisis line sector. Specifically, this review aims to answer the following research questions: (1) what AI use cases have been developed, designed, implemented, or evaluated in a crisis line context? (2) how, and to what degree, are equity, ethical, safety, and privacy challenges addressed or operationalized in AI applications for crisis lines? and (3) how do different interest holders (eg, service providers, service users, people with lived and living experience, and policymakers) in the crisis sector perceive and engage with AI?

## Methods

### Study Design

This scoping review will be conducted in accordance with the methodological guidance for scoping reviews proposed by Arksey and O’Malley [26] and the PRISMA-ScR (Preferred Reporting Items for Systematic Reviews and Meta-Analyses extension for Scoping Reviews) [27]. The final scoping review manuscript will also be reported in accordance with the PRISMA-ScR reporting guideline.

### Protocol Registration

This review was registered through the Open Science Framework website on March 16, 2026.

### Eligibility Criteria

Eligibility criteria will be defined using the population, concept, context framework [28].

#### Population

Studies will not be excluded based on population. To be eligible, studies must describe the use of AI in the crisis line sector. Studies describing the perspectives of different interest holders (eg, policymakers, service providers, service users, and people with lived and living experience) on the application of AI will be included.

#### Concept

The inclusion criteria are based on 2 key concepts: the application of AI technologies and crisis line services.

#### Learning-Based AI

For the purpose of this review, AI includes any use of learning-based AI models for analysis, decision support, quality monitoring, training, risk prediction, or other functions related to crisis lines. To ensure a comprehensive review of AI applications in the crisis sector, we include studies applying ML, deep learning, NLP, LLMs, and/or RAG models. We aim to include primary research that describes the development, testing, piloting, implementation, or evaluation of learning-based AI use cases in the crisis line sector.

We will exclude studies that describe applications of rule-based systems only, as much of the innovation and related ethical concerns are associated with learning-based and generative AI models [29-31]. We will include all learning-based models (eg, recommender and retriever-based models) so that we can assess the degree to which the crisis sector has, or has not, kept pace with AI innovations.

#### Crisis Lines and Mental Health Helplines

Crisis lines or mental health support lines are defined as nonprofit services with a mandate to address acute mental health concerns, including suicidal thoughts and behaviors, through synchronous interactions via text, chat, or phone. Crisis line services focused on mental health and crisis support typically have the capacity to receive and immediately respond to incoming calls, texts, or chats. Crisis lines are often available 24/7 at no cost to a regional public (eg, national and municipal). This may include national suicide prevention crisis lines or community specific helplines with a mental health support mandate. Crisis lines and mental health helplines typically have governing policies, principles, and documents (eg, terms of use and standard operating procedures) and require service providers (eg, crisis line responders) to undergo training. Crisis line responders often vary in their professional backgrounds and level of expertise (eg, from university students to trained therapists) and may fulfill their role as paid employees or as volunteers or a combination of both. We include crisis lines that provide peer support, provided other elements are present. Quit lines are excluded given their narrow focus.

Studies describing mental health chatbots, asynchronous mental health apps, or conversational AI agents (eg, ChatGPT) responding to mental health crises that are unaffiliated with an existing crisis line or mental health helpline will be excluded.

#### Context

Studies conducted in any country that are available in English will be included.

These criteria are intended to capture primary empirical research that directly examines real-world or proposed uses of AI within crisis line settings, while excluding literature unrelated to crisis lines or AI applications.

### Limitations and Restrictions

We will include peer-reviewed published literature (eg, journal articles, peer-reviewed book chapters, and peer-reviewed conference proceedings that provide detailed information and are identified through the search strategy) and gray literature (ie, dissertations, protocols, and trial registrations) describing primary research, with no publication date limits. The review will exclude conference abstracts, position papers, expert opinion pieces, editorials, and commentaries as these are unlikely to contain meaningful information. Papers on governance, policy, ethics, and legal considerations will also be excluded as our primary interest is in understanding how, and to what extent, ethics, policy, and governance considerations are integrated (or not) into efforts to apply AI to the crisis line context. Primary research represents the most appropriate evidence for addressing our research questions. Therefore, papers on governance, policy, ethics, and legal considerations that do not report primary research are outside the scope of this review. Eligibility criteria are summarized in Table 1.

**Table 1:** Summary of the eligibility criteria for the scoping review.

	Inclusion criteria	Exclusion criteria
Source type	Peer-reviewed journal articles, book chapters, conference proceedings, dissertations, protocols, and trial registrations	Conference abstracts, position papers, expert opinion pieces, editorials, and commentaries
Content	Primary research describing AI applications to crisis lines or helplines or interest holder views on AI applications in the crisis line sector	Primary research describing chatbots, AI agents, or asynchronous mental health apps that are unaffiliated with a crisis line or helpline Literature on governance, policy, ethics, and legal considerations that does not describe primary research on AI applications to crisis lines or helplines
Methods	Any	None
Other	Available in English	Not available in English

### Handling Ambiguous Information

All team members will review and discuss the eligibility criteria prior to screening to ensure a shared interpretation of inclusion and exclusion requirements.

If eligibility is unclear based on available information, the study will be retained for full-text review. Ambiguous cases will be discussed among team members until consensus is reached. If consensus cannot be reached, a senior analyst will make the final determination.

### Information Sources and Search Strategy

A search strategy was developed in collaboration with a medical research librarian (MH). Seven electronic databases were searched: Ovid MEDLINE, APA PsycInfo, Embase, Web of Science, IEEE Xplore, ACM Digital Library, and CINAHL. We searched a combination of medical databases, psychology databases, interdisciplinary databases, nursing databases, and a subject-specific (technology) database to ensure broad coverage. The review team screened sample results retrieved by draft searches, which aided the librarian in refining the search strategy. No date limit or language filters were applied. All searches were run on Thursday, March 12, 2026. The search strategy consists of 2 concepts combined using Boolean operators: (1) crisis lines (eg, hotlines, helplines, and chatlines) and (2) AI (eg, ChatGPT, LLMs, and NLP). Each concept was searched using a combination of subject headings, natural language keywords, and advanced search operators (eg, truncation, adjacency modifiers, and exact phrases). Animal studies were excluded from the search. All searches will be rerun closer to the completion of the manuscript to capture any new publications. The full search strategy (Ovid MEDLINE) is presented in Multimedia Appendix 1. Gray literature searches were also conducted in Preprints.org and ClinicalTrials.gov. Additional gray literature searches were run in Google to capture relevant organization reports, preprints, and government documents. The gray literature search strings are available upon request.

All retrieved citations will be imported into Covidence systematic review software. Once uploaded into Covidence, duplicates will be removed. Screening and review processes will also be conducted using Covidence. Additionally, reviews identified through the search strategy that are relevant to the concepts of interest will be hand-searched to identify additional articles that meet the inclusion criteria. The hand-search process will involve reviewing reference lists and associated abstracts to identify sources meeting the eligibility criteria. Review papers will be tracked, and eligibility determinations from the hand-search process will be documented.

### Study Selection

#### Title and Abstract Screening

All retrieved records will undergo title and abstract screening. A pilot screening exercise will first be conducted on a random sample of studies to ensure consistent application of criteria. Reviewers will independently screen titles and abstracts in duplicate in Covidence. To help guide decision-making, a screening tool will be developed and pilot-tested for use by the screening team (refer to Multimedia Appendix 2 for the draft screening tool). Discrepancies will be resolved through weekly consensus meetings. When eligibility is unclear at this stage, records will be advanced to full-text review.

#### Full-Text Screening

Full-text articles deemed potentially relevant will be independently reviewed in duplicate by the review team. A pilot phase will be conducted to refine understanding of the criteria. Disagreements will be resolved through weekly discussions. Reasons for exclusion at the full-text stage will be recorded in Covidence.

### Data Charting

A comprehensive data extraction form has been developed to capture relevant information from included studies (refer to Multimedia Appendix 3 for the draft data extraction form). The form is designed to collect key study characteristics, descriptions of crisis line services, and details related to the use or application of AI technologies. To facilitate the extraction and synthesis of information related to AI use cases, the form includes sections for tracking AI type, specific models used, data sources, functional roles, target end user, system autonomy (autonomous vs human-in-the-loop ), performance scores where available, and intended purpose. Additional sections have been included to capture information on implementation stage and readiness based on the AI life cycle, including whether the AI use case is in the development, piloting, or implementation stage [32].

To further address our research questions, we will use the readiness evaluation for AI-mental health deployment and implementation (READI) framework [22] to guide data extraction and synthesis. The READI framework was created to better capture the unique implications of AI applications in mental health care. Guided by the principles of minimizing harm and maximizing benefit, the framework outlines 6 key components that should be considered when developing and implementing AI tools in mental health contexts: safety, privacy, equity, clinical effectiveness, engagement, and implementation. Transparency underlies many of these components but will be captured separately in this review. Other concepts of interest concerning ethics, privacy, and equity will also be captured in this form (eg, trust, fairness, and transparency) [16]. The data extraction form will be pilot-tested for further refinement prior to use.

Data extraction will be conducted independently by reviewers using the data extraction form. A pilot extraction phase will be completed to ensure consistency. Completed forms will be reviewed and validated by a second team member. Discrepancies will be resolved through weekly discussions. Studies reporting on the same dataset (“companion studies”) will be identified and handled together to avoid duplication of data.

### Synthesis and Presentation of Results

Extracted data will be reviewed and cleaned for accuracy and consistency prior to analysis. Data management and cleaning will be conducted using Microsoft Excel.

A PRISMA (Preferred Reporting Items for Systematic Reviews and Meta-Analyses) flow diagram will be used to illustrate the study selection process, including numbers of records identified, screened, excluded, and included.

Findings will be synthesized using descriptive and narrative approaches. Results will be organized to map key characteristics of the literature, including study designs, types of AI applications, crisis line contexts, reported perspectives of interest holders, and the degree to which studies discuss the components outlined in the READI framework. A deductive content analysis [33] will be conducted in which each extracted data item will be summarized to describe how AI is being applied in crisis services across the READI framework components and other concepts of interest. Data will be presented in tables, figures, and diagrams as appropriate to summarize trends and gaps.

### Ethical Considerations

As this study involves the review and synthesis of gray and published literature, research ethics board approval is not required.

### Dissemination

The research team will oversee all stages of the project, with an experienced librarian contributing to search strategy development and methodological support. Findings from the review will be disseminated through peer-reviewed publications and presented at relevant academic and professional conferences. Results may also be shared with interest holders involved in crisis line services and AI applications to inform future research and practice. All reporting will adhere to established guidelines for scoping reviews to ensure transparency and methodological rigor.

## Results

**Figure 1:**
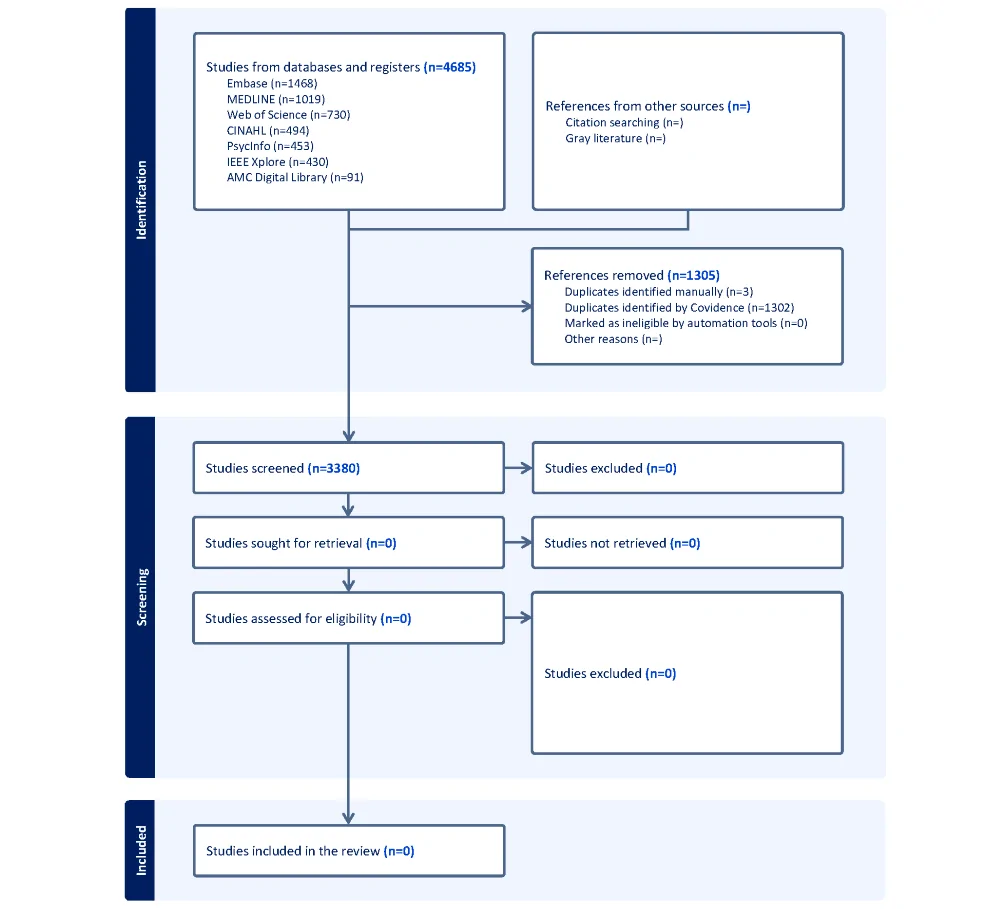
PRISMA (Preferred Reporting Items for Systematic Reviews and Meta-Analyses) flow diagram of the scoping review process.

The search strategy was finalized and executed across 7 databases in April 2026. This search yielded a total of 4685 records. Following the removal of 1305 duplicates, 3380 unique studies remain for screening (Figure 1). As of June 2026, screening is ongoing and is expected to conclude by September 2026. Results from this review are expected to be available by winter 2027.

## Discussion

This scoping review will provide a comprehensive map of how AI is being conceptualized, implemented, and evaluated within the crisis line sector. By systematically identifying and characterizing AI use cases across different modalities (eg, phone, text, and chat) and jurisdictions, the review will directly address our first research question regarding what AI applications have been developed or proposed for crisis lines and what implementation strategies have been used to support them. Synthesizing this information will identify which aspects of the crisis line workflow (eg, triage, risk assessment, quality monitoring, training, supervision, documentation, and follow-up) are currently the focus of AI innovation and which remain underexplored.

Using the READI framework as an organizing lens will enable us to examine how safety, privacy, equity, effectiveness, engagement, and implementation are addressed in the existing literature. This approach will address our second research question by clarifying the extent to which current or proposed AI applications for crisis lines explicitly consider these domains and identifying where reporting is limited or absent. For instance, we anticipate that technical performance (eg, model accuracy) may be better described than issues such as equity or long-term implementation, reflecting broader trends in the AI and mental health literature [21]. Mapping these gaps will help identify priority areas for future empirical work, such as the need for rigorous evaluation of AI impacts on user outcomes, responder workflow, and service-level indicators, as well as more systematic consideration of harms, unintended consequences, and mitigation strategies.

This review will also synthesize evidence on interest holder perspectives, including those of crisis line responders, service users, people with lived and living experience, policymakers, and technology developers. Prior work has highlighted ambivalence and concern about the potential for AI to undermine authenticity, trust, and the human relationship that is central to crisis support [12,14]. By collating available qualitative and quantitative evidence on perceptions, acceptability, and engagement, the review will address our third research question and provide a more nuanced understanding of how different groups perceive risks and opportunities. In particular, examining the extent and nature of co-design approaches will clarify whether and how people with lived and living experience, crisis line responders, and other interest holders are involved in the development and deployment of AI tools and whether participatory methods influence the design or uptake of these technologies.

More broadly, this review responds to several key gaps in the literature. First, although AI in mental health and suicide prevention has been the subject of growing research, existing syntheses have largely focused on prediction models or clinical settings rather than crisis helplines specifically [6,7,21]. To our knowledge, there is no existing review that concentrates on AI applications in real-time crisis support environments, where immediacy, safety, and relational care are paramount. Second, there is limited consolidation of evidence about how AI is currently being integrated into routine crisis line practice vs remaining at the conceptual or pilot stage. Differentiating between developed, tested, and deployed systems will help clarify the current state of implementation readiness in this sector.

Third, equity, privacy, and ethical considerations are often discussed in conceptual or policy-oriented papers but are less frequently examined in empirical studies. For example, a scoping review on LLM applications to suicide prevention accordingly found that ethical considerations were only discussed in a third of reviewed studies [21]. By restricting our review to primary research, we aim to identify where these issues are being operationalized in real-world or test-bed contexts and where empirical evidence is lacking. For example, the review may reveal a paucity of studies examining how AI-enabled crisis line tools perform across different populations, languages, or cultural contexts or how data governance, transparency, and informed consent are handled in practice. This will provide an evidence-informed basis for future work focused on equitable and rights-based AI deployment in crisis services.

The findings of this review are expected to have practical implications for crisis line organizations, policymakers, and technology developers. For crisis line leaders and practitioners, the review will offer an overview of existing and emerging AI tools, their reported benefits and challenges, and the conditions under which they have been implemented. This can inform decisions about whether and how to adopt AI solutions, what safeguards and governance structures are necessary, and which use cases (eg, training, workflow support, and quality improvement) may be most appropriate for early integration. For policymakers and regulators, the synthesized evidence on safety, privacy, and equity will help identify regulatory priorities and highlight areas where guidance is currently insufficient. For developers, the review may underscore the importance of co-design, transparency, and ongoing evaluation within the highly sensitive context of crisis support.

This review also has anticipated limitations. Restricting inclusion to English-language publications may omit relevant work from non–English-speaking contexts, where crisis services and AI development may be evolving differently. The focus on primary empirical research means that conceptual and policy perspectives will not be captured systematically, although these may be important for understanding the broader ethical and governance landscape. Additionally, the rapid pace of AI development, particularly in generative AI and LLMs, means that new applications may emerge during or after completion of the review. We will mitigate this by documenting the search dates clearly and, where feasible, incorporating recent gray literature that meets our inclusion criteria.

Despite these limitations, this review is timely. National crisis lines such as Canada’s 9-8-8 and other helplines worldwide are actively exploring ways to enhance capacity, consistency, and quality of care, often under significant resource constraints. As AI becomes more prevalent in public mental health and digital health infrastructures, crisis lines face increasing pressure to evaluate and potentially adopt these tools. By systematically mapping current AI applications; interest holder perspectives; and how core domains such as safety, privacy, and equity are addressed, this review will provide a foundational evidence base to guide responsible, person-centered AI integration in the crisis sector. It will also clarify where evidence is weakest, thereby informing a future research agenda focused on rigorous evaluation, participatory design, and implementation science approaches tailored to crisis line contexts.

## Appendix

Multimedia Appendix 1 Search strategy.

Multimedia Appendix 2 Screening tool version 2.

Multimedia Appendix 3 Data charting template version 3.

Multimedia Appendix 4 PRISMA ScR Checklist.

## Abbreviations

LLM: large language model
ML: machine learning
NLP: natural language processing
PRISMA: Preferred Reporting Items for Systematic Reviews and Meta-Analyses
PRISMA-ScR: Preferred Reporting Items for Systematic Reviews and Meta-Analyses extension for Scoping Reviews
RAG: retrieval-augmented generation
READI: readiness evaluation for AI-mental health deployment and implementation

## Data Availability

The datasets generated or analyzed during this study are available from the corresponding author on reasonable request.
