# Supplementary material for: Exploring Applications of AI in the Crisis Line Sector: Protocol for a Scoping Review
Source: JMIR Res Protoc. 2026 Aug 13;15:e95087. doi: 10.2196/95087 (PMC13522811; doi:10.2196/95087)
Supplement: Supplementary file 1 [file resprot_v15i1e95087_app1.docx]

**Exploring Applications of Artificial Intelligence in the Crisis Line Sector: Protocol for a Scoping Review**

Scoping Review Search Strategy

Ovid MEDLINE(R) ALL <1946 to March 11, 2026>

1	Hotlines/

2	Call Centers/

3	exp Telephone/ and exp Suicide/

4	Crisis Intervention/

5	Suicide Prevention/

6	exp Emergency Services, Psychiatric/

7	Mental Health Teletherapy/

8	hotline*.ab,hw,kf,ti.

9	hot-line*.ab,hw,kf,ti.

10	helpline*.ab,hw,kf,ti.

11	help-line*.ab,hw,kf,ti.

12	cris?s line*.ab,hw,kf,ti.

13	support line*.ab,hw,kf,ti.

14	assistance line*.ab,hw,kf,ti.

15	wellness line*.ab,hw,kf,ti.

16	mental health line*.ab,hw,kf,ti.

17	psych* line*.ab,hw,kf,ti.

18	care line*.ab,hw,kf,ti.

19	emergenc* line*.ab,hw,kf,ti.

20	chatline*.ab,hw,kf,ti.

21	chat-line*.ab,hw,kf,ti.

22	call centre*.ab,hw,kf,ti.

23	call center*.ab,hw,kf,ti.

24	cris?s text*.ab,hw,kf,ti.

25	cris?s chat*.ab,hw,kf,ti.

26	suicid* chat*.ab,hw,kf,ti.

27	(internet* adj3 chat*).ab,hw,kf,ti.

28	(web adj3 base* adj3 chat*).ab,hw,kf,ti.

29	9-8-8.ti,ab.

30	"988".ti,ab. and (suicid* or cris?s or mental health*).ab,hw,kf,ti.

31	warmline*.ab,hw,kf,ti.

32	warm-line*.ab,hw,kf,ti.

33	((((cris?s or ((emotional* or mental* or psychological*) adj1 distress*) or suicid*) adj3 (center* or centre* or counsel* or helper* or intervene* or operator* or mentor* or peer* or personnel* or program* or responder* or service* or staff* or supervisor* or support* or team* or trainer* or worker* or workforce* or volunteer*)) and (caller* or chat* or line or lines or messag* or phone$1 or phoning* or telephon* or text* or warmline* or warm-line*)) or ((SMS or MMS) adj5 messag*)).ab,hw,kf,ti.

34	((telephone* or cellphone* or phone* or text* or SMS or call or calls or caller or callers) adj5 (suicid* or selfharm* or self-harm* or cris?s or self innjur* or self mutilat* or parasuicid* or automutilat* or auto-mutilat* or ((psych* or emotional* or mental*) adj3 distress*))).ab,hw,kf,ti.

35	((critical or cris?s) adj3 triag*).ab,hw,kf,ti.

36	(suicid* adj1 (prevent* or cris?s or support*) adj1 (line* or hotline* or helpline*)).ab,hw,kf,ti.

37	(lifeline* or life-line*).ab,hw,kf,ti.

38	or/1-37 [**Crisis lines/hotlines/helplines/etc.]

39	exp Artificial Intelligence/

40	algorithms/

41	autoencoder/

42	Data Mining/

43	(artificial* adj3 intelligence*).ab,hw,kf,ti,id,jw,kw.

44	(artificial* adj3 superintelligence*).ab,hw,kf,ti,id,jw,kw.

45	(large adj3 language* adj3 model*).ab,hw,kf,ti.

46	(machine* adj3 learn*).ab,hw,kf,ti.

47	(deep* adj3 learn*).ab,hw,kf,ti.

48	(natural adj3 language* adj3 processing).ab,hw,kf,ti.

49	(natural adj3 language* adj3 processor*).ab,hw,kf,ti.

50	(conversational adj3 agent*).ab,hw,kf,ti.

51	(chatbot* or chat-bot*).ab,hw,kf,ti.

52	(intelligent* adj3 system*).ab,hw,kf,ti.

53	(comput* adj3 intelligence*).ab,hw,kf,ti.

54	(digital* adj3 intelligence*).ab,hw,kf,ti.

55	((predict* adj3 model*) and (AI or artificial intelligence*)).ab,hw,kf,ti.

56	(ChatGPT or Chat-GPT).ab,hw,kf,ti.

57	(Gemini and (Google or Bard or artificial intelligence* or AI)).ab,hw,kf,ti.

58	(generat* adj3 AI).ab,hw,kf,ti.

59	(blackbox* adj3 AI).ab,hw,kf,ti.

60	(black-box* adj3 AI).ab,hw,kf,ti.

61	(general adj3 purpose* adj3 AI).ab,hw,kf,ti.

62	AGI.ab,hw,kf,ti.

63	(AGI and (artificial intelligence* or AI)).ab,hw,kf,ti.

64	critical AI.ab,hw,kf,ti.

65	(AI adj3 hallucinat*).ab,hw,kf,ti.

66	explainable AI.ab,hw,kf,ti.

67	(XAI and AI).ti,ab.

68	neural network*.ab,hw,kf,ti.

69	(ANN and artificial neural network*).ab,hw,kf,ti.

70	intelligence augmentation.ab,hw,kf,ti.

71	(Copilot and (microsoft or artificial intelligence* or AI)).ab,hw,kf,ti.

72	Character AI.ab,hw,kf,ti.

73	Perplexity AI.ab,hw,kf,ti.

74	Replika.ab,hw,kf,ti.

75	(Elicit and (artificial intelligence* or AI)).ab,hw,kf,ti.

76	OpenAI.ab,hw,kf,ti.

77	(AI and (artificial intelligence* or large language model* or deep learning or machine learning)).ab,hw,kf,ti.

78	(LLM and (artificial intelligence* or large language model* or deep learning or machine learning)).ab,hw,kf,ti.

79	(ML and (artificial intelligence* or large language model* or deep learning or machine learning)).ab,hw,kf,ti.

80	(NLP and (artificial intelligence* or large language model* or deep learning or machine learning or natural language processing)).ab,hw,kf,ti.

81	genAI.ab,hw,kf,ti.

82	gen-AI.ab,hw,kf,ti.

83	(AI adj3 model*).ab,hw,kf,ti.

84	(AI adj3 literac*).ab,hw,kf,ti.

85	(AI adj3 ethic*).ab,hw,kf,ti.

86	(generat* adj3 pretrain* adj3 transform*).ab,hw,kf,ti.

87	(generat* adj3 pre-train* adj3 transform*).ab,hw,kf,ti.

88	affective comput*.ab,hw,kf,ti.

89	association rule min*.ab,hw,kf,ti.

90	data min*.ab,hw,kf,ti.

91	algorithm*.ab,hw,kf,ti.

92	autoencoder*.ab,hw,kf,ti.

93	or/39-92 [**Artificial intelligence]

94	and/38,93 [**Crisis lines and AI]

95	(Animals/ or Models, Animal/ or Disease Models, Animal/) not Humans/

96	((animal or animals or canine* or dog or dogs or feline or hamster* or lamb or lambs or mice or monkey or monkeys or mouse or murine or pig or pigs or piglet* or porcine or primate* or rabbit* or rats or rat or rodent* or sheep* or veterinar*) not (human* or patient*)).ti,kf,jw.

97	or/95-96 [**Animal studies]

98	94 not 97 [**Excluding animal studies]
