# Supplementary material for: Exploring Applications of AI in the Crisis Line Sector: Protocol for a Scoping Review
Source: JMIR Res Protoc. 2026 Aug 13;15:e95087. doi: 10.2196/95087 (PMC13522811; doi:10.2196/95087)
Supplement: Supplementary file 2 [file resprot_v15i1e95087_app2.docx]

**Exclude**

Is this a peer-reviewed article, conference proceeding, book, dissertation, report, registered protocol, registered trial chapter available in English?

No

**Exclude**

Does it describe crisis sector interest holder perspectives (e.g., service users, responders, providers) on AI?

Yes

**Include!**

No

Yes

**Include!**

Yes

Does it describe the application of AI (e.g., LLMs, machine learning, NLP, generative AI, and/or deep learning)?

No

**Exclude**

No

Yes

Does it describe a crisis line with a mental health mandate?
